# Research on Intelligent Identification and Grading of Nonmetallic Inclusions in Steels Based on Deep Learning

**DOI:** 10.3390/mi14020482

**Published:** 2023-02-19

**Authors:** Xiaolin Zhu, Wenhai Wan, Ling Qian, Yu Cai, Xiang Chen, Pingze Zhang, Guanxi Huang, Bo Liu, Qiang Yao, Shaoyuan Li, Zhengjun Yao

**Affiliations:** 1 College of Material Science and Technology, Nanjing University of Aeronautics and Astronautics, Nanjing 211106, China; 2 Jiangsu Product Quality Testing & Inspection Institute, Nanjing 210007, China; 3 Jiangsu Changbao Precision Steel Tube Co., Ltd., Changzhou 213200, China; 4 Jiangsu Zhongxin Pipe Sci-Tec Co., Ltd., Nanjing 211100, China

**Keywords:** non-metallic inclusions, segmentation and classification, intelligent rating, deep learning

## Abstract

Non-metallic inclusions are unavoidable defects in steel, and their type, quantity, size, and distribution have a great impact on the quality of steel. At present, non-metallic inclusions are mainly detected manually, which features high work intensity, low efficiency, proneness to misjudgment, and low consistency of results. In this paper, based on deep neural network algorithm, a small number of manually labeled, low-resolution metallographic images collected by optical microscopes are used as the dataset for intelligent boundary extraction, classification, and rating of non-metallic inclusions. The training datasets are cropped into those containing only a single non-metallic inclusion to reduce the interference of background information and improve the accuracy. To deal with the unbalanced distribution of each category of inclusions, the reweighting cross entropy loss and focal loss are respectively used as the category prediction loss and boundary prediction loss of the DeepLabv3+ semantic segmentation model. Finally, the length and width of the minimum enclosing rectangle of the segmented inclusions are measured to calculate the grade of inclusions. The resulting accuracy is 90.34% in segmentation and 90.35% in classification. As is verified, the model-based rating results are consistent with those of manual labeling. For a single sample, the detection time is reduced from 30 min to 15 s, significantly improving the detection efficiency.

## 1. Introduction

Nonmetallic inclusions in steel refer to oxides and silicates formed by a small quantity of slags, refractories, and deoxidation products during steel smelting, as well as sulfides and nitrides formed due to the declining of solubility of some elements (such as sulfur and nitrogen) during steel solidification. These compounds cannot be timely discharged and thus remain in steel as inclusions. Under particular circumstances, such inclusions may be beneficial to certain properties of steel; for example, they can improve the machinability of steel [1]. However, they are generally harmful to the properties of steel. Inclusions exist in steel as independent phases, which destroy the continuity [2,3] of the steel’s matrix, increase the inhomogeneity of thesteel’sstructure, and lower the mechanical properties of steel, especially the plasticity, toughness, and fatigue life. This leads to aremarkably declined quality and performance of steel products. Therefore, nonmetallic inclusionsare an important indicator to measure the quality of steel. Their type, quantity, shape, size, distribution, and other factors have an impact on steel properties. For high-quality special steel, nonmetallic inclusions are listed as a compulsoryinspection item.

Nonmetallic inclusions are divided into four categories by their shape and distribution: Group A (sulfide type): highly malleable, individual gray particles with a wide range of aspect ratios (length/width), and generally rounded ends; Group B (alumina type): numerous non-deformable, angular, low aspect ratio (generally < 3), black or bluish particles (at least three) aligned in the deformation direction; Group C (silicate type):highly malleable, individual black or dark gray particles with a wide range of aspect ratios (generally > 3) and generally sharp ends; Group D (globular oxide type): circular or nearly circular, individual particles; plus the subcategory Group DS (single globular type): single particle with a diameter of 13 μm and above. Detection of nonmetallic inclusions is to observe a 200 mm^2^ polished specimen with a microscope, whose field of view is 0.5 mm^2^ under working conditions, that is, 400 fields of view should be observed for each sample, and the category of inclusions in each field of view should be judged and their length measured. For each category of inclusions, the result of its worst field of view is taken as the final result [4].

At present, non-metallic inclusions mainly rely on manual detection. This involves extremely high workintensity, and can easily cause visual fatigue and affect the health of personnel due to long-term work in the field of view with a high-power microscope. In addition, the identification and classification of nonmetallic inclusions require better professional background and practical experience since inexperienced personnel are prone to misjudgment. Therefore, manual detection of nonmetallic inclusions has obvious shortcomings, such as high work intensity, low detection efficiency, easy misjudgment, and low consistency of results.

With the rapid development of electronic information technology and artificial intelligence technology, computer vision (CV), a discipline that studies how to make computers “see” like humans, is widely used in various industries, such as text recognition, and medical diagnosis [5,6]. In the field of materials, the automatic metallographic analysis of metal materials based on computer vision and machine learning technologies has become the focus of researchers at home and abroad. Qin [7] introduced a dependable and efficient way to establish the relationship between composition and detrimental phases in Ni-base superalloys by machine learning algorithms. Long [8] utilizeda random forest algorithm on the magnetic ground state, and the Curie temperature (TC)to classify ferromagnetic and antiferromagnetic compounds and to predict the TC of the ferromagnets. In Kaufmann’s paper [9], a high-throughput CNN-based approach to classifying electron backscatter diffraction patterns at the space group level is developed and demonstrated. Patxi [10] reconstructed the austenite grain boundary in martensitic steel using the deep learning algorithm. Wang [11] applied computer vision and machine learning to identify where fatigue cracks begin in materials. Azimi [12] employed pixel-wise segmentation based on full convolutional neural networks (FCNN), together with the deep learning method to classify and identify the microstructure of mild steel, achieving 93.94% classification accuracy. In Li’s paper [13], Gradient boosting decision trees wereused to recognize the boundaries of a lath bainitic microstructure. The optimized machine learning model achieved greater than 88% recognition accuracy for all boundaries in the bainitic microstructure.

Han [14] proposed a high-throughput characterization method based on deep learning, rapid acquisition techniques, and mathematical statistics to identify, segment, and quantify the microstructure of weathering steel. The segmentation accuracies of 89.95% and 90.86% for non-metallic inclusions and pearlite phases, respectively, and the detection time are significantly reduced. Han used a large number (more than 120,000 images) of high-resolution SEM images as training dataset. However, in the actual work of non-metallic inclusions detection, the optical microscope is used for observation and measurement at 100×, and the resolution of the optical microscope is much lower than that of the SEM images. Also, Han only detected one of the inclusions and the grade of the inclusions was not rated. Thus Han’s paper provides a good method for intelligent identification and segmentation of microstructures, but the practicality of his intelligent grading work for non-metallic inclusions is still a long way off and needs further research.

Therefore, this paper explores the employing of computer vision and deep learning technologies in identifying and detecting nonmetallic inclusions, in order to improve the detection efficiency and avoid manual detection errors. Specifically, in this paper, based on deep neural network image segmentation algorithm, a small number (2000 images) of manually labeled, low-resolution metallographic images collected by optical microscopes are used as the dataset to train the model. Then, five types of non-metallic inclusions are classified and boundary extracted simultaneously, so as to achieve intelligent rating of non-metallic inclusions.

## 2. Preparatory Work

### 2.1. Image Acquisition

A bearing steel bar with a diameter of 50 mm was used as the testing raw material. A total of 20 pieces of 20 mm × 10 mm metallographic samples were randomly cut, ground, and polished, and then manually observed and photographed using the ZEISS optical microscope. In total, 2000 low-resolution metallographic photographs with non-metallic inclusions were collected.

### 2.2. Image Calibration

**Figure 1:**
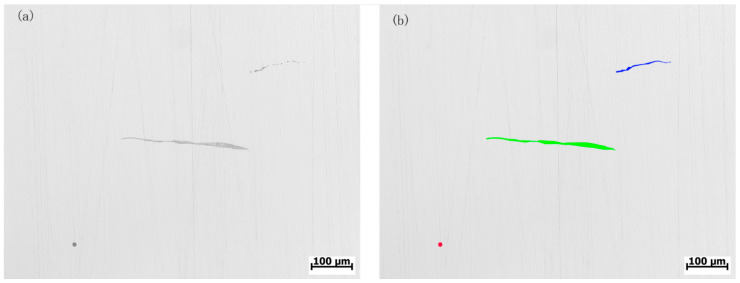
Original metallograph (**a**) and its labeling result (**b**).

With the help of the LabelMe platform, metal slice images were distributed to human experts for determining the categories, boundaries, and grades of inclusions in accordance with the standard [4,15] for detection of nonmetallic inclusions. Figure 1 shows an example of labeling a single metal slice image and the boundary of its inclusion. In this paper, 1864 valid metallographic images (2048 × 1536) and their labeling files were collected.

## 3. Construction of the Model

### 3.1. Network Architecture

**Figure 2:**
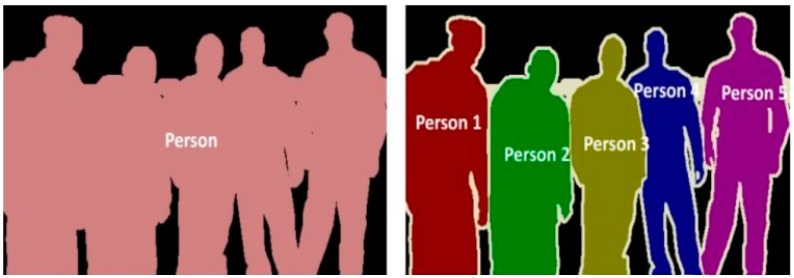
Comparison of semantic segmentation (**left**) and instance segmentation (**right**).

Image segmentation, an important field of computer vision, is about segmenting different content entities in scene images into different image regions [16]. Image segmentation includes semantic segmentation and instance segmentation. The formerrequires the assignment of category labels to different image regions while the latter requires further classification of different object instances within the category. Figure 2 shows the specific forms of semantic segmentation and instance segmentation taking the human image as an example. In this paper, theidentification and grading of nonmetallic inclusions is in essence the identification of the boundaries and categories of inclusions, which, as a result, can be modeled as the semantic segmentation of images.

Conventional semantic segmentation methods, such as the commonly used classical segmentation methods based on thresholds, regions and edges, mainly classify the edge, color, texture, and other features in the image. The threshold-based image segmentation algorithm is used to predict a threshold by the gray-scale value of the whole image, and compare it with the pixel in the image for the purpose of pixel division. The region-based segmentation method is used to identify different regions based on the uniformity of a region in the image. The image segmentation technology based on edge detection is used to make segmentation depending on the gray scale change of a pixel on the edge of the image: the pixel that has a dramatic change to itsgray scaleis the boundary point. The image can be divided into multiple target regions by finding each pixel located on the boundary and connecting all boundary points.

In this paper, based on the DeepLabv3+ semantic segmentation network, a loss function is designed specific to the data distribution characteristics of nonmetallic inclusions for high-precision boundary extraction and classification of inclusions. DeepLabv3+generated a new Encoder-Decoder model based on DeepLabv3, which extracted and fused rich context information, and improved the edge detection accuracy of each target image.

**Figure 3:**
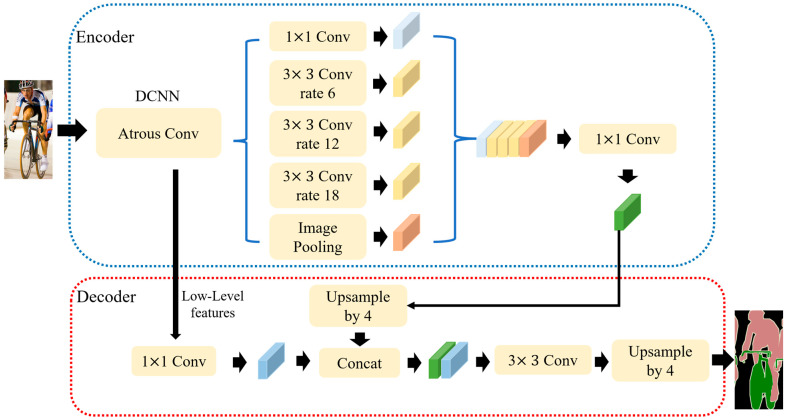
Schematic diagram of DeepLabv3+ network architecture. Adapted from [17].

DeepLabv3+ network architecture [17], as shown in Figure 3, uses DeepLabv3 as an Encoder to extract and fuse multi-scale features; and uses the Decoder for further fusion of low-level features and high-level features to improve the accuracy of the segmentation boundaries and to create more details, thus forming a new network fusing Atrous Spatial Pyramid Pooling (ASPP) and Encoder-Decoder.

The Atrous convolution module in the encoder, a core DeepLab model and a convolution method to increase receptive fields, helps to extract multi-scale information. It has “holes” added on the basis of ordinary convolution, and uses the dilation rate to control the size of the receptive field. Due to the screen door effect of Atrous convolution, the image through Atrous convolutional computation will lose some information. Therefore, in order to reduce the probability of information loss and improve the network accuracy, the encoder remedies the defects of Atrous convolution through ASPP, and uses Atrous convolution with different expansion rates to capture multilevel context and fuse all results obtained.

### 3.2. Loss Function

This section discusses the loss function used in the algorithm, which is used to evaluate the difference between the model predictions and the actual results. Different loss functions will affect the final optimization results of model parameters. Usually, the more rational the loss function is designed to be, the better the performance of the model.

The inclusion training data labeled is represented by $D=\left(X,Y,\;Y_{s}\right)$, where $X={\left\{x_{i}\right\}}_{i=1}^{N}$ is the inclusion image set; $Y={\left\{y_{i}\right\}}_{i=1}^{N}$ corresponds to the category label set of the inclusion; $Y_{s}={\left\{y_{is}\right\}}_{i=1}^{N}$ corresponds to the boundary label of the inclusion; the i-th training image is represented by $x_{i}$; the category label and the boundary label of the i-th image are represented by $y_{i}$ and $y_{is}$, respectively, and N is the number of images. For the inclusion problem to be solved in this paper, there are 6 possible values for category label, i.e., 5 inclusion categories and the background which is not an inclusion in the image.

As defined by the above symbols, the loss function (L(D)) in this paper is comprised of category prediction loss ($l_{1}$) and boundary prediction loss ($l_{2}$):

(1) $$L\left(D\right)=\overset{N}{\underset{i=1}{\sum }}\left[l_{1}\left(h^{1}\left(x_{i}\right),y_{i}\right)+l_{2}\left(h^{2}\left(x_{i}\right),y_{is}\right)\right]$$

where, $l_{1}$ is the category loss predicted and $l_{2}$ is the boundary loss predicted; $h^{1}\left(x_{i}\right)$ and $h^{2}\left(x_{i}\right)$ represent the category label prediction and the boundary label prediction, respectively, of image $x_{i}$ by semantic segmentation model h.

The metallographic images mostly consists of a background part, with only a small percentage of inclusions. Different categories of inclusions are not in balanced distribution. To prevent under-fitting of tail-categories and over-fitting of head-categories by the model, this paper uses Reweighting Cross Entropy Loss and Focal Loss as the category prediction loss and the boundary prediction loss, respectively, which will be detailed below.

Reweighting Cross EntropyLoss:Cross entropy loss (CE Loss) is used to determine the proximity between the predicted and the expected outputs of the model. For sample x with category labeled as y, the cross entropy $H\left(h^{1}\left(x\right),y\right)$ between the model category predicted $\;h^{1}\left(x\right)$ and its real category y can be expressed by the following formula:

(2) $$H\left(h^{1}\left(x\right),y\right)=-\underset{c}{\sum }\left[y_{c}log\;h_{c}^{1}\left(x\right)\right]$$

where, $h_{c}^{1}\left(x\right)$ is the predicted probability of the model against category c, and $y_{c}$ is the true probability of category $c.H\left(h^{1}\left(x\right),y\right)$ describes the difference between the predicted probability distribution $h^{1}\left(x\right)$ and the real category $y_{c}$: the smaller the cross entropy, the higher the proximity between two probability distributions and the better the model prediction.

To deal with the category imbalance, this paper introduces weight into the loss of each category, i.e.,:

(3) $$l_{1}\left(h^{1}\left(x\right),y\right)=-\underset{c}{\sum }w_{c}\left[y_{c}log\;h_{c}^{1}\left(x\right)\right]$$

where, $w_{c\;}$ is the weight of customized category c, which is defined below:

(4) $$w_{c}=\frac{n_{max}}{n_{c}}$$

where, $n_{max}$ is the number of samples corresponding to the largest category, and $n_{c}$ is the number of samples of category c.

Focal Loss:It should be noted that in semantic segmentation, the sample boundary label is represented by the category label set of all pixels in the image, i.e., for sample x with boundary labeled as $y_{s}$, $y_{s}={\left\{y_{p}\right\}}_{p=1}^{np}$, where $y_{p}$ is the category label of pixel $x_{p}$ in the image, and np is the number of pixels in the image. The boundary prediction loss $l_{2}\left(h^{2}\left(x\right),y_{s}\right)$ of the model can be expressed as the sum of the category label prediction losses of all pixels, where, Focal Loss [18] is adopted as the loss $l\left(h^{2}\left(x_{p}\right),y_{p}\right)$ on each pixel $x_{p}$. Parameter γ is added on the basis of cross entropy lossto create a regulatory factor to solve the problem of sample imbalance. The formula for calculation of loss function is detailed as follows:

(5) $$l\left(h^{2}\left(x_{p}\right),y_{p}\right)=-\underset{c}{\sum }y_{pc}{\left(1-h_{c}^{2}\left(x_{p}\right)\right)}^{\gamma }log\;h_{c}^{2}\left(x_{p}\right)$$

It can be seen that a regulatory factor ${\left(1-h_{c}^{2}\left(x_{p}\right)\right)}^{\gamma }$ is added for the model prediction $h_{c}^{2}\left(x_{p}\right)$ on the basis of cross entropy loss, and γ is a parameter. When $y_{pc}=1\;a$ nd the model predicted value $h_{c}^{2}\left(x_{p}\right)$ is approximate to 1, i.e., when the model prediction is accurate, the regulatory factor ${\left(1-h_{c}^{2}\left(x_{p}\right)\right)}^{\gamma }$ is approximate to 0. Thus the focal loss is reduced in comparison with the cross entropy loss; when $y_{pc}=1$ and the model predicted value $h_{c}^{2}\left(x_{p}\right)$ is approximate to 0, i.e.,; when the model prediction is inaccurate, the regulatory factor ${\left(1-h_{c}^{2}\left(x_{p}\right)\right)}^{\gamma }$ is approximate to 1. The focal loss remains unchanged in comparison with the cross entropy loss. Therefore, when the data categories are unbalanced, focal loss is equivalent to the increased loss weight of the subcategory data, which tends to be predicted incorrectly on the basis of cross entropy loss.

## 4. Experiment

### 4.1. Data Preprocessing

The non-metallic inclusions in steel have an extremely low content, and cover a very small area in the original metallographic image, leaving large blank areas that do not contain inclusionsin the images. In addition, there are interference items, such as scratches and contamination, in the original images. To minimize the requirement on computer performance and interference with the training model, the training datasetsare cut out according to the labeled json data file by following the principles below:

(1) The area cut out contains only a single nonmetallic inclusion; if there are multiple inclusions, the classification accuracy of the model and the subsequent grading of inclusions will be affected.
(2) The edge length of the cut area is determined by the inclusion size. Since inclusions in some images are undersized and direct cutting according to the boundary will blur the original image and the labeled image, it is necessary to set the minimum length and width in cutting.

A total of 4785 metallographic images, each containing a single nonmetallic inclusion have been produced after the original images were cut. See Table 1 for the distribution of each category.

### 4.2. Training Setting

As shown in Figure 1, the original metallographic images are of low quality, with low resolution and blurred inclusions boundaries. In this paper, the preprocessed metallographic images are used as datasets that are randomly divided into training datasets and testing datasets based on a proportion of 9:1; ResNet [19] is selected as the backbone network for feature extraction; by introducing residual neurons, ResNet can transmit some signals to the output end directly, without the use of a convolutional network. This helps retain lower-level information, thus avoiding the loss of feature details because of deepened feature networks. The model in this paper is based on the PyTorchframework, using Python as the programming language and NVIDIA GeForce RTX 3090 as GPU.

It is necessary to optimize parameters in the process of model training. The training set parameters mainly include batch size; the training parameters mainly include the learning rate and epoch; and the model parameters include the input size and output size. The batch size is the number of samples in one iteration, and different batch sizes will affect the time required to complete each round of learning and the smoothness of gradient between iterations. If the batch size is too small, the training process will take a longer time, resulting in greater gradient oscillation, which is not conducive to convergence. If the batch size is too large, the model is likely to get stuck in a local minimum. The epoch is the total number of training rounds required for the training data in one training, and an epoch represents training all data for one round. The learning rate is the parameter of using the loss function gradient to adjust the network weight in the gradient-descent algorithm. A learning rate too high will result in unstable learning, while a learning rate too low will consume too much time during training. Therefore, choosing a proper learning rate can generate better performance with the gradient-descent algorithm. Parameters are optimized using the methods specified in the literature and on the basis of empirical value. The learning rate of the model used in this paper is eventually set as 0.001, the batch size as 128, and the epoch as 100.

### 4.3. Evaluation Indicators

In this paper, the segmentation and classification of nonmetallic inclusions in metallographic images are realized through the deep learning network model. The evaluation indicators include the mean pixel accuracy (mPA) and the mean intersection over union (MIoU)commonly used in semantic segmentation, as well asaccuracycommonly used in classification. The meanings and calculation formulas of these evaluation indicators will be introduced in the confusion matrix below.

**Figure 4:**
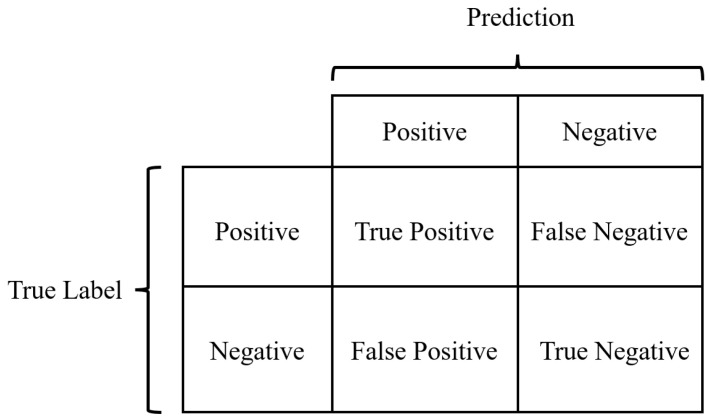
Schematic diagram of the confusion matrix for the binary classification task.

The confusion matrix is used to visualize the predicted classification result and the real classification result of the model. Each column of the confusion matrix represents the predicted category, and each row represents the real category of data. Figure 4 shows the confusion matrix under the binary classification task.

For a binary classification task, the model prediction result may fall into the following four types:

TP (True Positive): means that a sample is predicted to be positive and labeled positive.

FN (False Negative): means that a sample is predictedto benegative, but labeled positive.

FP (False Positive): means that a sample is predicted to be positive, but labeled negative.

TN (True Negative): means that a sample is predicted to be negativeand labeled negative.

Pixel Accuracy (PA): means the percentage of pixel values predicted correctly, which is calculated as below:

(6) $$PA=\frac{TP+TN}{TP+FP+FN+TN}$$

mPA is the mean value derived from the sum of pixel accuracy of each category.

IoU is the intersection-to-unionratio between the predicted result of a certain category and the true label, which is calculated as below:

(7) $$IoU=\frac{TP}{TP+FP+FN}$$

MIoU is the mean value derived from the sum of IoUs of each category.

Accuracy is represented by the percentage of sample sizes correctly predicted, corresponding to the pixel accuracy in segmentation. Its calculation formula is the same as that of (6).

### 4.4. Result and Discussion

**Figure 5:**
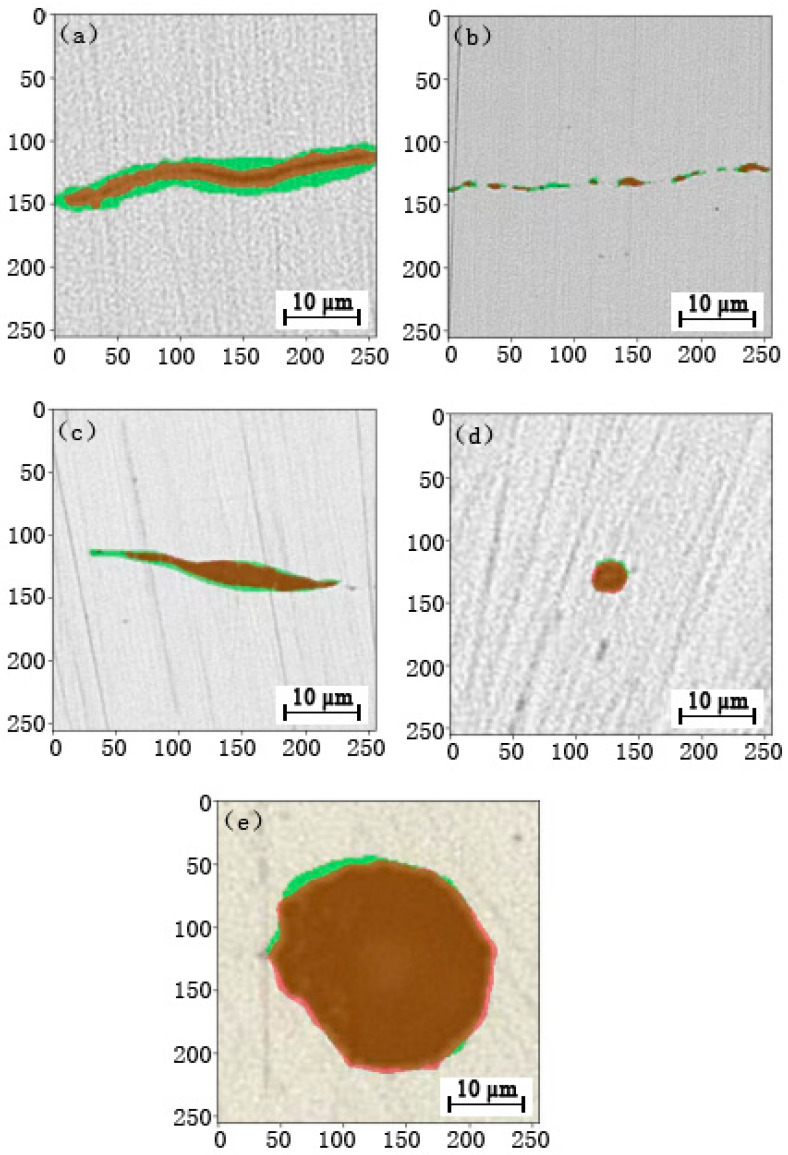
Diagram of segmentation effect of category A (**a**), B (**b**), C (**c**), D (**d**), and DS (**e**).

In this experiment, DeepLabv3+ semantic segmentation model and ResNet34 are used as the backbone networks for training, and the output after the training superimposes the original image, labeled image, and prediction boundary. The final visualization effects are shown as below:

Figure 5a–e show the boundary extraction effects of five categories of inclusions, with the green part indicating the boundary of manually labeled nonmetallic inclusions, and the red part indicating the boundary of model-predicted metallic inclusions. It can be seen that this model generates better segmentation effects, with a boundary more accurate than thatlabeled manually. The accuracy of thenonmetallic inclusion segmentation finally achieved with the semantic segmentation model based on DeepLabv3+ is 90.34%, and the accuracy of nonmetallic inclusion classification achieved is 90.35%. See Table 2 below for detailed results. The segmentation method used in this paper is significantly better than conventional ones.

Defects, such as scratches, contaminants and holes, do exist in the original metallographic samples, which are interference terms for the training and prediction of deep learning models. This paper uses three methods to solve to improve the accuracy of non-metallic inclusions prediction. Firstly, fine polishing and protection of the samples reduces the number of defects in the samples. Secondly, accurate calibration of the images: scratches, contaminants and holes, and inclusions are different in shape and color, such as scratches are mainly long straight lines, while contaminants and holes are different from inclusions in gray scale or shape. The calibration of non-metallic inclusions is done by experienced metallographic analysis experts who do not mark these interfering terms as inclusions. Accurate calibration will ensure that the model will be less likely to identify the interfering items in the sample as inclusions in prediction. Thirdly, thealgorithm, which understands that interfering items differ from the inclusions in size, grayscale, or shape, is able to avoid the misclassification of these differences. For example, in size, the minimum width of A, B, and C inclusions is 2 μm, and the minimum diameter of D inclusions is 3 μm. After the inclusions are identified by the model, we exclude strip-shaped items below 2 μm and round items below 3 μm from the automatic rating process. In grayscale, the inclusions are gray or nearly black. Furthermore, we were able to set grayscale thresholds that exclude colors that are too light or too dark, which are generally interfering terms, such as contaminants, holes, or corrosive water stains.

**Figure 6:**
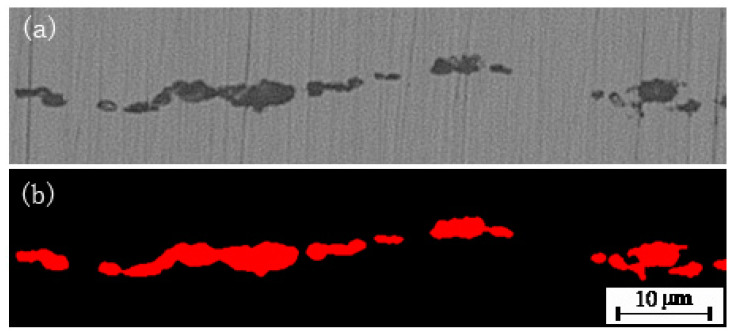
Image of nonmetallic inclusions: (**a**) original, (**b**) segmented by the model.

**Figure 7:**
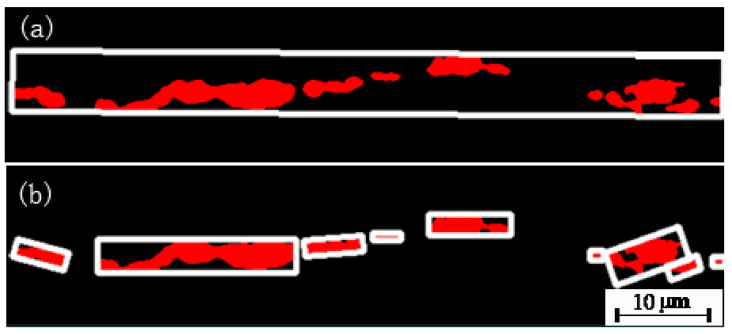
Establishing the length (**a**) and width (**b**) by forming the minimum bounding rectangle.

Figure 6a shows the original inclusions and Figure 6b shows the inclusions segmented by this model, both of which match very well. The grading of nonmetallic inclusions is to calculate the length and width of the segmented boundary of nonmetallic inclusions by revoking the image processing function in OpenCV to the acquire the minimum enclosing rectangle formed by the boundary of nonmetallic inclusions, and calculating the grade of nonmetallic inclusions according to the length and width, thus minimum the enclosing rectangle. The length refers to the long side of the minimum enclosing rectangle formed by nonmetallic inclusions as a whole, and the width refers to the statistical width of the largest block of all nonmetallic inclusions, for which the minimum enclosing rectangle is formed, as shown Figure 7.

In Figure 7a above, the minimum enclosing rectangle is formed for the nonmetallic inclusion as a whole to obtain its true length of 66.2 µm through conversion on the scale, and the inclusions fall into Category B Grade 0.5. In Figure 7b, the minimum enclosing rectangle is formed partially for the nonmetallic inclusion to obtain its true maximum width of 3.14 µm through conversion on the scale, and the inclusions fall into Subcategory B. For a single sample, the detection time is reduced from 30 min to 15 s, significantly improving the detection efficiency.

After the effectiveness of the DeepLabv3+ semantic segmentation model is verified, comparison experiments were conducted on different backbone networks. According to the evaluation indicators in Table 3 below, there is only a small discrepancy between the precision of segmentationand classification of nonmetallic inclusions in this model, both are around 90%; in terms of the training speed, the DeepLabv3+ semantic segmentation model is faster.

## 5. Conclusions

In this paper, based on the deep neural network algorithm, manually labeled low-quality metallographic images are collected as training samples for intelligent boundary extraction, classification, and grading of nonmetallic inclusions. To highlight information about inclusions and minimize the interference of background information, image datasets containing only a single nonmetallic inclusion are acquired by cutting the original metallographic images in the data preprocessing stage. To deal with the unbalanced distribution of all categories of inclusions, the segmentation and classification loss functions are designed for the DeepLabv3+ semantic segmentation model, realizing the segmentation and classification of nonmetallic inclusions. Finally, the length and width are calculated based on the minimum enclosing rectangle formed by segmented inclusion boundaries for grading. The resulting accuracy is 90.34% in segmentation and 90.35% in classification. As is verified, the model-based rating results are consistent with those of manual labeling. For a single sample, the detection time is reduced from 30 min to 15 s, significantly improving the detection efficiency. This method has realized the intelligent identification and rating of non-metallic inclusions for the first time, greatly reducing such problems as big errors, subjective influence, and poor consistency, which tend to occur in manual inspection. In the future, improvements will focus on the following aspects. Firstly, the datasets in this paper are small in quantity and the data of each category are unbalanced; in the future, dataset extension methods such as data enhancement will be used to improve data quality and model effects. Secondly, this paper only focuses on the processing of pre-processed images containing a single nonmetallic inclusion; in future studies, attempts will be made to realize fast and efficient identification and grading of images containing multiple nonmetallic inclusions. Finally the method in this paper will be further applied to the identification of other complex grain patterns to truly realize intelligent automatic metallographic analysis and promote the development of metallographic analysis.

## Figures and Tables

**Table 1:** Distribution of image categories of nonmetallic inclusions after cutting.

Category	Quantity	Percentage
A	689	0.144
B	831	0.174
C	310	0.065
D	2792	0.583
DS	163	0.034

**Table 2:** Model Training Result.

Seg_Model	Backbone	mPA	MIoU	Acc.	Epoch Time (s)
DeepLabv3+	ResNet34	90.34%	76.90%	90.35%	9

**Table 3:** Horizontal comparison experiment results.

Seg_Model	Backbone	mPA	MIoU	Acc.	Epoch Time (s)
DeepLabv3+	ResNet34	90.34%	76.90%	90.35%	9
DeepLabv3+	ResNet50	88.19%	78.01%	91.67%	9

## Data Availability

Not Applicable.
